# Rapid destruction of the humeral head due to bone fragility: report of two cases

**DOI:** 10.1007/s00776-012-0290-0

**Published:** 2012-08-31

**Authors:** Katsuhisa Yoshikawa, Kazuya Tamai, Yuichiro Yano, Nakayuki Kato, Miwa Akutsu, Kazuo Tomizawa, Yutaka Nohara

**Affiliations:** Department of Orthopedic Surgery, Dokkyo Medical University School of Medicine, 880 kitakobayashi, Mibu, Tochigi, 321-0293 Japan

## Introduction

Rapid destruction of the femoral head has been well understood since Postel’s report in 1970 [1]. This condition occurs mostly in elderly women, and considerable concern has been directed to the subchondral insufficiency fracture as a cause of femoral head collapse [2]. In contrast, rapid destruction of the humeral head is rare; only one case of nonnecrotic humeral head collapse is reported in the literature [3]. We treated two patients whose humeral head collapse was most possibly due to an insufficiency fracture. The patients and/or their families were informed that data from the case would be submitted for publication and gave their consent.

### Case 1

**Fig. 1:**
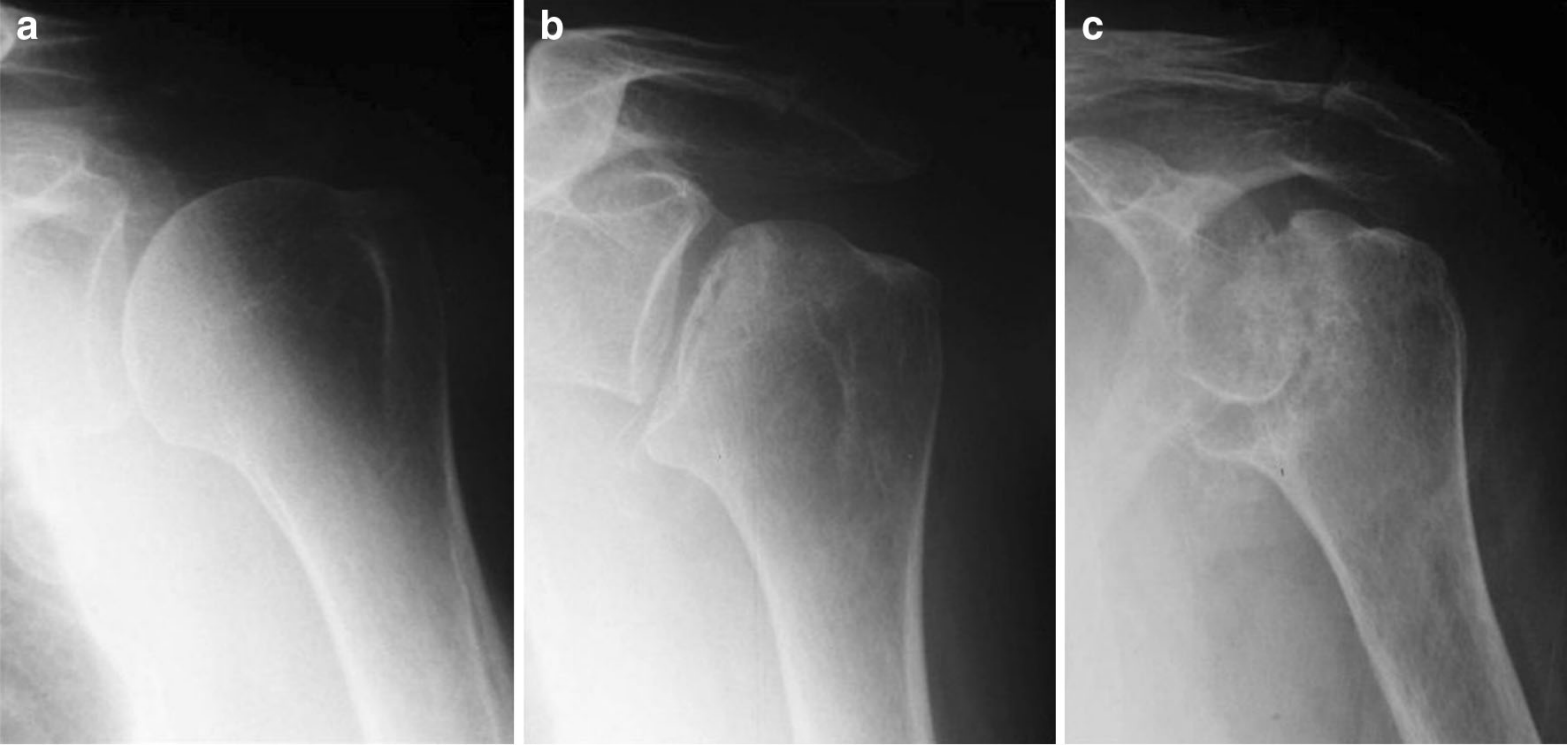
Serial preoperative radiographs of case 1. No abnormalities were noted 3 weeks after the injury (**a**). However, 8 weeks after the injury, the humeral head showed a slight degree of collapse (**b**), which became significant 8 months after the injury (**c**)

A 74-year-old woman suffered left shoulder pain following a fall on her left hand from a standing position during farm work. She also had difficulty elevating the arm 3 weeks after the fall. X-ray of her left shoulder taken at that time looked normal (Fig. 1a). The patient consulted with a second orthopedic surgeon 8 weeks after the fall, when some collapse of the humeral head was found on X-ray (Fig. 1b). An intra-articular injection of a mixture of local anesthetic and corticosteroid did not improve her symptoms. As the humeral head collapse appeared worse on X-ray taken 8 months after the fall (Fig. 1c), she was referred to us for further treatment. Her medical and family history were unremarkable.

**Fig. 2:**
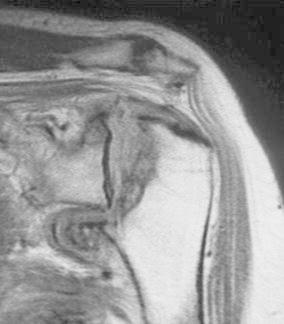
Preoperative magnetic resonance images (MRI) of case 1. In addition to effusion, a low signal area was noted in the humeral head in proton-weighted imaging. The rotator cuff seemed intact

On examination, the left shoulder was not swollen but showed slight muscle atrophy. Active range-of-motion of the left shoulder were 90° in flexion, 20° in external rotation at the side, and S2 in internal rotation posteriorly. There were no neurologic abnormalities. Blood tests proved normal; urine amino-terminal collagen cross-links (NTx) was 88.7 (normal 9.3–54.3) nmol bone collagen equivalents (BCE)/mmol creatinine, and urine deoxypyridinoline was 12.8 (normal 2.8–7.6) nmol/mmol creatinine. Dual-photon absorptiometry using Hologic-2000 showed reduced bone mineral density (BMD) (74 % for the lumbar spine, 69 % for the proximal femur, 58 % for the distal radius) compared with the young adult mean (YAM). Magnetic resonance imaging (MRI) revealed effusion in the glenohumeral joint and an intact rotator cuff. There was a low signal area in the humeral head in proton-weighted imaging (Fig. 2).

**Fig. 3:**
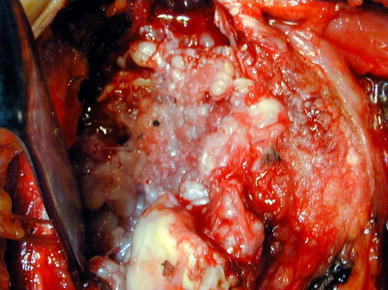
Operative findings of case 1. A deep notch was present in the center of the humeral head, which was covered with white, irregular scar tissues

**Fig. 4:**
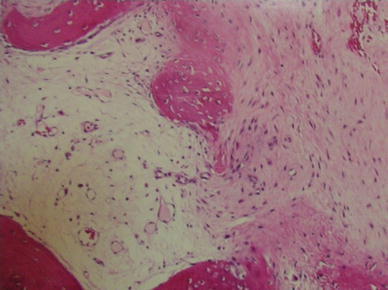
Microscopic findings of the humeral head in case 1. Neither fat necrosis nor dead bone was noted. There were lumps of granulation tissue in the subchondral area, with some broken trabeculae and callus formation. Hematoxylin and eosin (H&E), ×100

As a neuropathic arthropathy or infectious arthritis was denied by clinical and laboratory findings, we performed a hemiarthroplasty 9 months after the fall. At surgery, it was found that most of the articular cartilage had disappeared. A deep notch was present in the center of the humeral head, which was covered with white, irregular scar tissues (Fig. 3). On histologic examination, no evidence of osteonecrosis was noted in the humeral head. There were lumps of granulation tissue in the subchondral area, with some broken trabeculae and callus formation (Fig. 4).

**Fig. 5:**
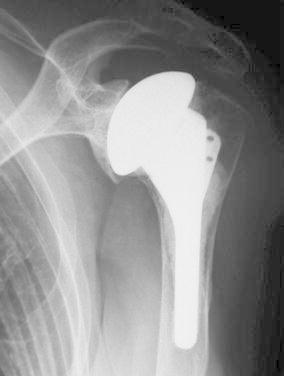
Postoperative radiographs of case 1. Six years after surgery, a slight degree of arthritic change was noted in the glenoid

Six years postoperatively, the patient was pain free, with no restrictions in activities of daily life, although the X-ray showed slight arthritic changes of the glenoid (Fig. 5).

### Case 2

**Fig. 6:**
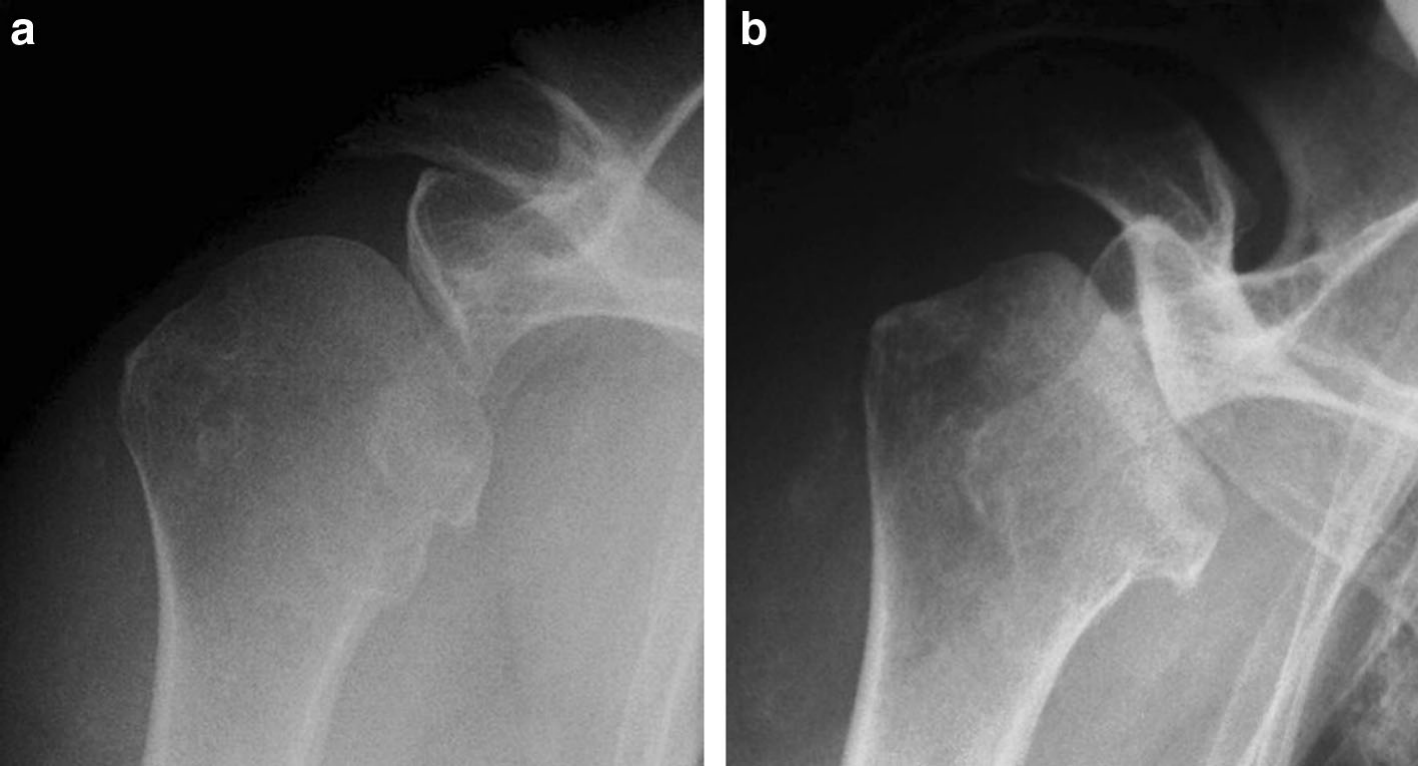
Serial preoperative radiographs of case 2. Flattening of the humeral head was noted 1 month after symptom onset (**a**), which became significant 2 months later (**b**)

**Fig. 7:**
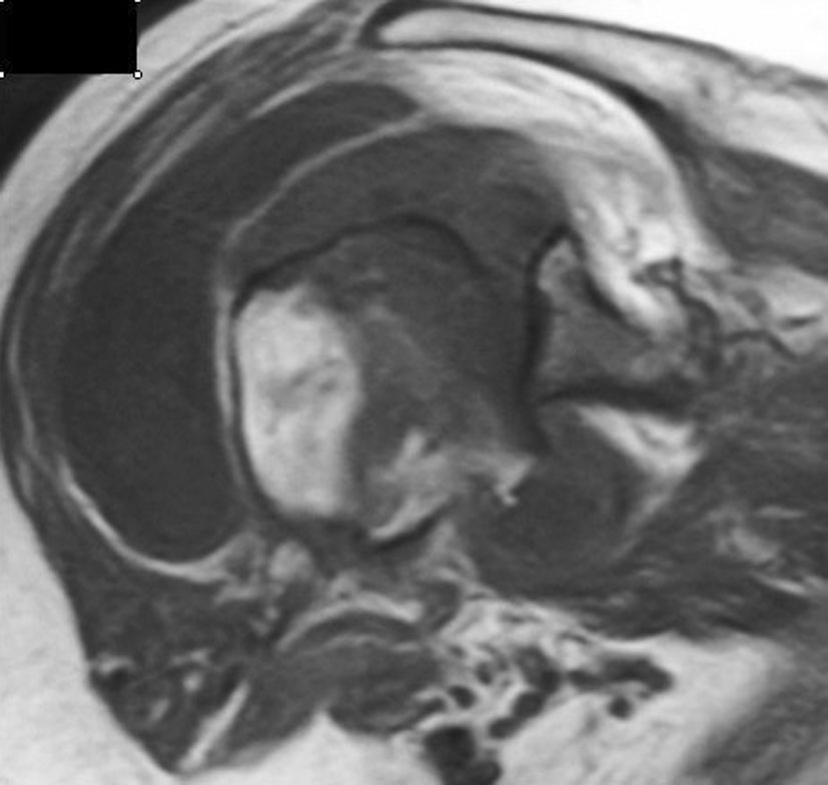
Preoperative magnetic resonance images of case 2. T1-weighted image shows the articular surface and center of the humeral head as low signal areas. The rotator cuff is preserved

A 78-year-old woman spontaneously developed right shoulder pain, for which she received medication from an orthopedic doctor. However, the pain did not improve, but rather, bone destruction was noted. She was referred to us 3 months after symptom onset. Medical history included diabetes mellitus, hypertension, bullous keratopathy, and osteoporosis. The patient had been treated with bisphosphonates and the active form of vitamin D_3_ for 3 years. On physical examination, there was tenderness over the anterior joint space of the right shoulder. Active ranges of motion were 75° in flexion, 15° in external rotation, and buttock in internal rotation posteriorly. No neurological abnormalities were noted. Blood tests showed no infectious disease or rheumatoid arthritis. Urine NTx was 20.3 (normal 9.3–54.3) nmol BCE/mmol creatinine, and urine deoxypyridinoline was 12.8 (normal 2.8–7.6) nmol/mmol creatinine. BMD (% YAM) was 97 % for the lumbar spine, 79 % for the proximal femur, and 83 % for the distal radius. The X-ray taken 1 month after symptom onset revealed a flattening of the humeral head. The humeral head collapse progressed during the next 2 months (Fig. 6). MRI showed effusion in the glenohumeral joint; the rotator cuff remained normal. T1-weighted imaging showed a low signal area stretching from the articular surface to the center of the humeral head (Fig. 7).

**Fig. 8:**
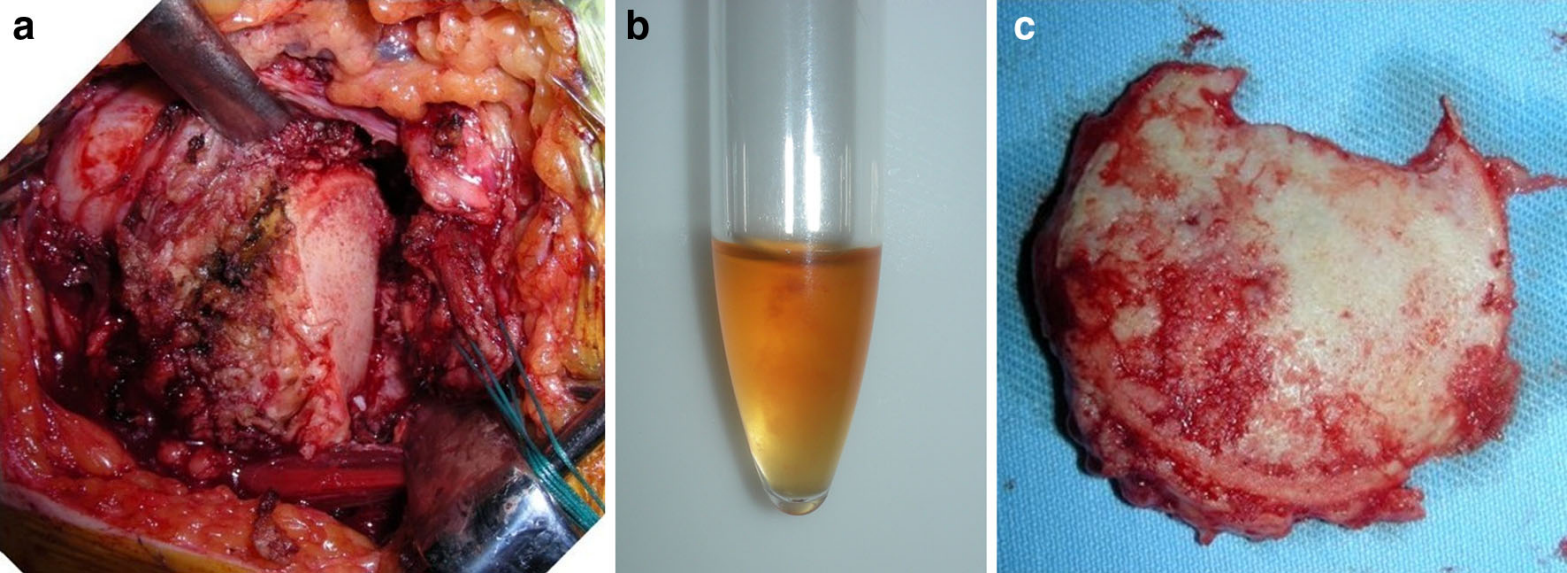
Operative findings of case 2. The articular cartilage was lost, and the subchondral bone was eburnated both in the glenoid (**a**) [a small amount of serobloody synovial fluid was seen in the glenohumeral joint (**b**)] and in the humeral head (**c**)

**Fig. 9:**
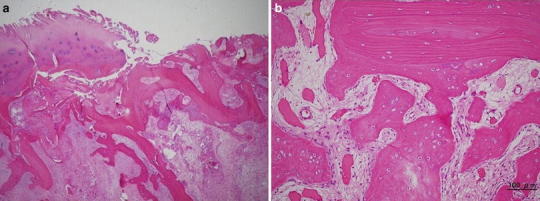
Microscopic findings of the humeral head in case 2. In some specimens, degenerated cartilage was noted (**a**) [hematoxylin and eosin (H&E) ×40]. There was abundant new bone formation surrounding preexisting trabeculae, which appeared near normal and did not suggest osteonecrosis (**b**) (H&E ×200)

As infection and neuropathic arthropathy were least likely, we implanted a total shoulder prosthesis 4 months after symptom onset. At surgery, the articular cartilage was lost and the subchondral bone was eburnated (Fig. 8). There was a small amount of serobloody synovial fluid in the glenohumeral joint, which proved not to contain any crystals on microscopic examination. Histologically, the articular cartilage was lost, although there was a small area where degenerated cartilage was noted. In the subchondral zone, there was abundant new bone formation but no evidence of osteonecrosis (Fig. 9).

**Fig. 10:**
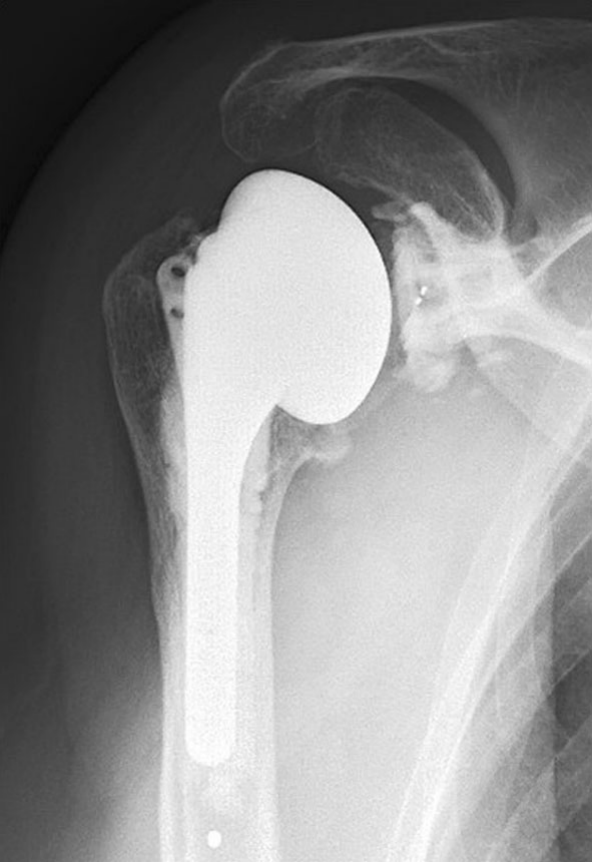
Postoperative radiographs of case 2. Thirty months after surgery, there was no hardware failure

Two and a half years postoperatively, the patient pain free, with no restrictions in activities of daily life (Fig. 10).

## Discussion

Nguyen reviewed 60 cases of rapid destructive arthritis of the shoulder and concluded that most cases were in elderly women and that this condition was characterized by the presence of a large amount of synovial fluid [4]. The two cases we report here apparently showed similar characteristics. Differential diagnoses of our cases include osteonecrosis of the humeral head, rheumatoid arthritis, pyogenic arthritis, crystal-induced arthritis, hemodialysis-related destructive arthropathy, osteoarthritis, steroid-induced arthropathy, and cuff-tear arthropathy. However, none of these conditions were likely considering the patients’ medical history, blood test results, synovial fluid findings, and imaging studies. In case 1, injection of corticosteroids may have affected the clinical course, but actually, the humeral head collapse was recognized before injection. As for case 2, although diabetes mellitus can cause neuropathic arthropathy, the diabetes was not severe, and deep sensations were normal in this patient.

In imaging studies, case 1 showed significant destruction of the humeral head on X-ray and a low-intensity area beneath the surface of the humeral head on MRI. Case 2 showed significant deformity of the humeral head with joint-space narrowing on X-ray and a low-intensity area on T1-weighted MRI and high-intensity area on T2-weighted images. The findings in case 1 are consistent with the stage 3 findings in rapidly destructive arthritis of the hip joint and those in case 2 with stage 2 findings [5]. Thus, the events that occurred in the humeral head of our patients can be similar to those in rapidly femoral head destruction.

It is known that the condition of the femoral articular cartilage varies in rapidly destructive arthritis of the hip joint. Yamamoto and Bullough [2] noted that the articular cartilage at the superior portion of the femoral head may be thinned, detached from the subchondral bone, or lost. The articular cartilage of the humeral head in case 2 of our report was lost, which was consistent with findings described by those authors. In addition, they and Mitrovic and Riera [6] observed granulomatous tissue in the marrow space, where there was no covering of articular cartilage, particularly in the advanced stage of rapidly destructive arthritis of the hip joint. This was consistent with granulomatous tissue formation in our case 1, in which surgery was carried out 9 months after symptom onset.

It should be noted that histologically there was new bone formation rather than osteonecrosis in the subchondral area in both cases. Tokuya et al. [3] reported a case of rapidly destructive arthrosis of the shoulder in which reactive new bone formation, consistent with fracture callus, was seen in the humeral head. The authors concluded that bone destruction in their case resulted from subchondral insufficiency fracture due to osteoporosis. This condition could favorably be compared with the rapidly destructive arthritis of the hip joint in which subchondral insufficiency fracture has been described as a cause of rapid joint destruction [2, 7, 8]. This was the case in our patients, who showed low BMD and elevated collagen metabolites. Thus, we conclude that humeral head destruction does occur as a result of insufficiency fracture in elderly women.
